# TREX1 as a Novel Immunotherapeutic Target

**DOI:** 10.3389/fimmu.2021.660184

**Published:** 2021-04-01

**Authors:** Wayne O. Hemphill, Sean R. Simpson, Mingyong Liu, Freddie R. Salsbury, Thomas Hollis, Jason M. Grayson, Fred W. Perrino

**Affiliations:** ^1^ Department of Biochemistry, Center for Structural Biology, Wake Forest School of Medicine, Winston-Salem, NC, United States; ^2^ Department of Microbiology and Immunology, Wake Forest School of Medicine, Winston-Salem, NC, United States; ^3^ Department of Physics, Wake Forest University, Winston-Salem, NC, United States

**Keywords:** exonuclease, small-molecule, inhibition, immunotherapy, cancer

## Abstract

Mutations in the TREX1 3’ → 5’ exonuclease are associated with a spectrum of autoimmune disease phenotypes in humans and mice. Failure to degrade DNA activates the cGAS-STING DNA-sensing pathway signaling a type-I interferon (IFN) response that ultimately drives immune system activation. TREX1 and the cGAS-STING DNA-sensing pathway have also been implicated in the tumor microenvironment, where TREX1 is proposed to degrade tumor-derived DNA that would otherwise activate cGAS-STING. If tumor-derived DNA were not degraded, the cGAS-STING pathway would be activated to promote IFN-dependent antitumor immunity. Thus, we hypothesize TREX1 exonuclease inhibition as a novel immunotherapeutic strategy. We present data demonstrating antitumor immunity in the TREX1 D18N mouse model and discuss theory surrounding the best strategy for TREX1 inhibition. Potential complications of TREX1 inhibition as a therapeutic strategy are also discussed.

## A Brief History of TREX1

**Figure 1:**
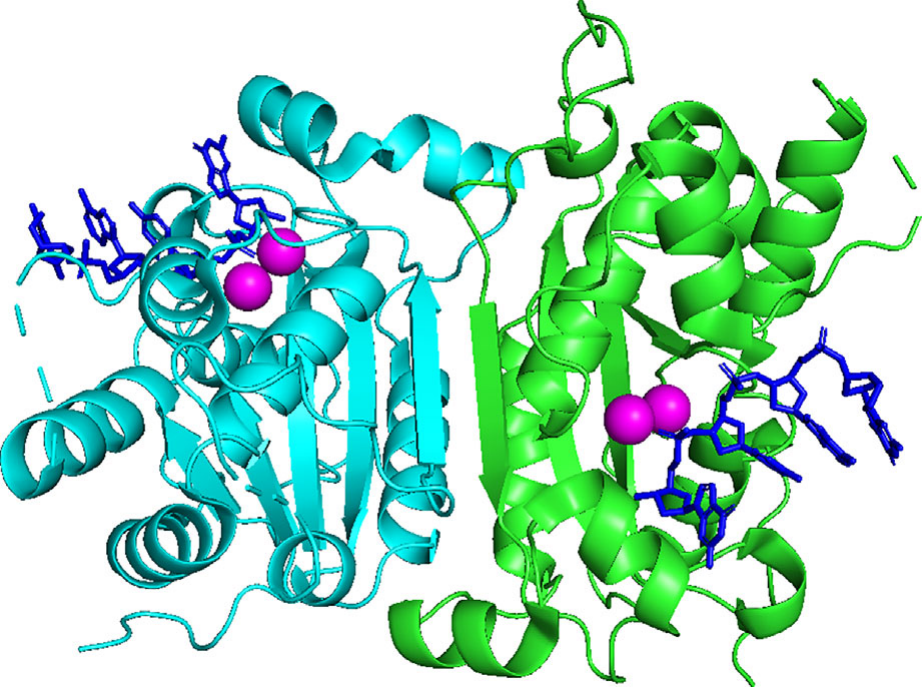
Crystal Structure of the Dimeric Exonuclease mTREX1(1-242). Structure includes only the TREX1 catalytic domain (1-242). Protomers are distinguished by green and cyan, ssDNA by blue sticks, and calcium ions by magenta coloring. Crystal structure was visualized in PyMOL using the PDB structure 2OA8 from ref (6).

Three-prime Repair EXonuclease 1 (TREX1) is a nonprocessive 3’ → 5’ exonuclease (1). Biochemical investigations of TREX1 established similar degradation activities using ss- and dsDNA substrates, with some preference for dsDNA with 3’-mismatches and 3’-overhangs. TREX1 activity using RNA and RNA-DNA duplexes is approximately 1000-fold less than with DNA, implicating DNA as the endogenous polynucleotide substrate (1–5). TREX1 is a 314 amino acid polypeptide composed of an N-terminal catalytic domain (1-242) containing the exonuclease activity (2), and a C-terminal region (243-314) (6) that facilitates localization of the enzyme to the perinuclear space in cells (7). The TREX1 C-terminal region has also been proposed to interact with the oligosaccharyltransferase (OST) complex (8, 9), and TREX1 has been proposed to function in the SET complex (10). TREX1 is a stable homodimer (**Figure 1**) with the protomers connected by an extended β-sheet core and a highly stable network of hydrogen bonds and hydrophobic interactions, such that the homodimer does not measurably dissociate after initial formation (11). The obligate dimeric structure of TREX1 is unique among exonucleases, and highly relevant to TREX1 catalytic activity. We have demonstrated that residues from one TREX1 protomer communicate across the dimer interface and contribute to catalysis in the opposing protomer, illustrating the requirement of TREX1’s dimeric structure for full exonuclease activity (12). These studies further suggest a potential mechanism for inter-protomer regulation and/or coordinated catalysis.

**Figure 2:**
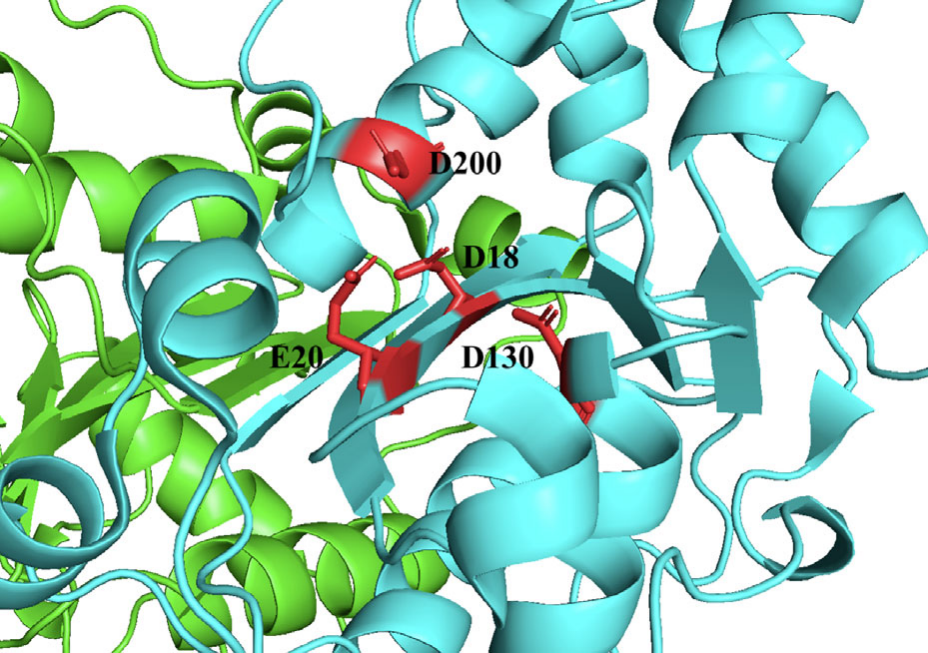
TREX1 is a Member of the DEDD Family of Exonucleases. Structure includes only the TREX1 catalytic domain (1-242). Protomers are distinguished by green and cyan cartoons, and D18-E20-D130-D200 motif residues are shown as red sticks with black labels. Crystal structure was visualized in PyMOL using the PDB structure 3MXJ from ref (16).

TREX1 is a member of the DEDD family of 3’ → 5’, whose members are defined by a conserved Asp-Glu-Asp-Asp motif that facilitates catalytic activity (13–15) (**Figure 2**). Members of the DEDD nuclease family frequently have a role in DNA replication and/or repair (1, 3, 17), prompting early investigations in this area for TREX1. However, mice lacking TREX1 do not develop a hyper-mutator phenotype, but instead develop an aggressive autoimmune phenotype characterized by inflammatory myocarditis (18). More than sixty TREX1 mutations have now been identified [reviewed in ref (19)] that exhibit dominant and recessive genetics and occur as inherited or *de novo* mutations, dependent upon the specific mutant allele. TREX1 disease alleles include missense mutations, insertions, duplications, and frame shifts that locate to positions throughout the 314-amino acid-coding gene. There is a causal relationship between TREX1 genetic variants and multiple mechanisms of TREX1 enzyme dysfunction that have now been linked to a spectrum of autoimmune diseases in humans (19). There is also some correlation between the positions of TREX1 mutations and the observed clinical phenotype. Most of the TREX1 mutations affecting the catalytic domain are recessive and are largely associated with Aicardi-Goutières Syndrome (AGS) or Familial Chilblains Lupus (FCL) (19). The dominant TREX1 mutations produce enzyme that competitively inhibits wild-type enzyme activity on bulky dsDNA substrates (11, 20–22). TREX1 mutations that cause Retinal Vasculopathy with Cerebral Leukodystrophy (RVCL) exhibit dominant inheritance and are exclusively frame-shift mutations in the C-terminal tail region of the enzyme (8, 19, 23). Additional frame-shift mutations in the C-terminal region result in recessive AGS (19). All together the TREX1 mutations indicate a complex relationship between TREX1 structure, function, genetics, and clinical disease.

A hallmark of TREX1 mutation is chronic type-I interferon (IFN) signaling. TREX1 deficient mice are completely rescued from mortality and pathology by introducing IFN receptor (IFNAR) deficiency, demonstrating that TREX1 disease pathology is driven by IFN signaling (24). Similar genetic studies have also demonstrated stimulator of interferon genes (STING) (25), interferon regulatory factor 3 (IRF3) (24), and cyclic GMP-AMP synthase (cGAS) (26–28) as critical components of the pathological mechanism, establishing the cGAS-STING DNA-sensing pathway’s role in TREX1 deficiency disease. In the cGAS-STING pathway, binding of dsDNA to cGAS causes synthesis of a 2’-3’-cyclic GMP-AMP (cGAMP) (29), which in turn binds to and activates the endoplasmic reticulum-associated protein STING (30, 31). Upon activation, STING traffics to the Golgi apparatus where it recruits Tank Binding Kinase 1 (TBK1) to phosphorylate it (32). Phosphorylated STING recruits IRF3 for phosphorylation by TBK1, and activated IRF3 then dimerizes and translocates to the nucleus to promote expression of IFN (33). After its expression, binding of IFN to IFNAR induces immune activation by promoting the proliferation and maintenance of natural killer (NK) and memory CD8+ T cells, stimulating dendritic cells (DC), and more broadly by increasing the expression of interferon-stimulated genes (ISGs) (34). The cGAS-STING pathway has been proposed to act as a broad sensing pathway for many sources of DNA (33). Collectively, studies to date support a model where deficiency in TREX1 exonuclease activity leads to accumulation of TREX1 DNA substrate(s), which then stimulate the cGAS-STING pathway and promote pathology *via* subsequent type-I IFN signaling.

TREX1 exonuclease dysfunction and subsequent cGAS-STING signaling raises questions about the source of immune activating DNA. Multiple sources of DNA have been proposed as potential sources of TREX1 substrate *in vivo*, including ssDNA replication intermediates (35), retroelements (24), and enucleated erythroblast DNA (36). Our lab (37) and others (38) have demonstrated that TREX1 inactivity in bone marrow-derived cells drives any discernable pathology, but other cells can contribute to IFN signaling. Ultimately, the question of TREX1 biological substrate(s) remains an area of active investigation. We have recently published a review of TREX1 (39), which we recommend for further details.

### TREX1 & cGAS-STING in the Tumor Microenvironment

TREX1 and the cGAS-STING pathway have been implicated in the tumor microenvironment [reviewed in refs (39–45)]. TREX1 activity has been negatively correlated with outcomes in multiple cancers (46–48). In addition, treatment of cancerous cells *in vitro* with UV-light or various genotoxic anti-cancer drugs is associated with *TREX1* upregulation, and siRNA knockdown of *TREX1* enhances cancer cell death following these treatments (49). A dose-dependent effect of DNA-damaging agents on TREX1 expression has been demonstrated, and showed that TREX1 degrades damaged DNA from drug-treated tumor cells (50). Finally, multiple studies have shown that the dose-dependent efficacy of radiotherapy is at least partially attributable to TREX1 activity (51–53).

IFN-dependent antitumor immunity following radiotherapy is STING-dependent, as demonstrated by its ablation in STING-deficient mice (54, 55). Additionally, cGAS-deficiency in DCs has been reported to be sufficient to abrogate antitumor immunity *in vitro* (54). However, there are additional studies that indicate IFN-production *in vitro* is unaffected by cGAS-knockout in DCs, but is attenuated by cGAS-knockout in tumor cells, by STING-knockout in DCs, or by connexin 43-knockout in tumor cells (56). These data led to the proposal that cGAS-mediated DNA-sensing is tumor-intrinsic, and that cGAMP, produced in tumor cells, is transferred *via* gap junctions to host DCs activating STING and initiating IFN-dependent antitumor immunity (56–59). Yet, additional work indicates that tumor-intrinsic and tumor-extrinsic STING participate in driving antitumor immunity (60). Thus, while current studies support cGAS-STING function in antitumor immunity following radiotherapy and/or chemotherapy, the precise nature of cGAS and STING’s roles in tumor and immune-cell function remain unresolved.

TREX1 is the gatekeeper enzyme of the cGAS-STING pathway, and tumor-derived DNA generated spontaneously or induced by radiotherapy or chemotherapy can be degraded by TREX1. DNA that is not degraded by tumor-intrinsic TREX1 can stimulate the cGAS-STING pathway to generate an IFN-response and drive immune cell recruitment to facilitate tumor regression. The initial cGAS-stimulation resulting from undegraded DNA could be tumor-intrinsic or immune cell-intrinsic with the resulting cGAMP signaling molecule transferred to neighboring cells. How tumor-derived DNA locates to the cytosol of immune cells remains unclear. Direct immune cell phagocytosis of tumor cells or exosome shuttling of tumor-derived DNA are possible, and the abundance of tumor-derived DNA correlates with tumor-intrinsic TREX1 expression (61). Thus, it is possible that cGAS-STING stimulation contributes to antitumor immunity in both tumor and host immune cells indicating that TREX1, cGAS, and STING are candidate targets to modulate antitumor immunity. Regardless, studies to date have demonstrated that TREX1, cGAS, and STING can be targeted to modulate antitumor immunity.

## cGAS and STING as Therapeutic Targets

TREX1 dysfunction activates the cGAS-STING DNA-sensing pathway resulting in autoimmunity. Thus, preventing cGAS-STING activation could provide therapeutic benefit to treat TREX1-mediated autoimmune disease. Inhibition of cGAS (62–65) and STING (66) using small molecules and anti-sense oligonucleotides have been shown to ameliorate pathology in mouse models of autoimmunity, and to limit brain injury following ischemic stroke (67). These studies support cGAS and STING as candidate targets for inhibition in autoimmune disease.

Conversely, stimulation of the cGAS-STING pathway is a novel approach to immune activation in cancer immune-therapy. Small-molecule STING agonists have been used to activate the cGAS-STING pathway and promote antitumor immunity (68–76). Additional work indicates STING agonists are effective in combinatorial therapies for infection (77). STING agonists are currently in clinical trials (78–80). DMXAA is a potent STING agonist that initially appeared promising in pre-clinical studies (70, 81, 82), but failed in human trials due to critical amino acid differences between the mouse and human STING proteins (83). These STING agonists indicate the potential in immunotherapy for cGAS-STING pathway activation. Since TREX1 exonuclease inactivity is known to stimulate cGAS-STING signaling, we propose TREX1 inhibition as an anticancer immunotherapeutic strategy.

## TREX1 Inhibition as an Immunotherapeutic Strategy

The molecular and cellular properties of TREX1 indicate it has distinct advantages as a molecular target for immune activation. Studies indicating TREX1 expression is induced by genotoxic stress and that TREX1 exonuclease activity protects cancer cells from anticancer drugs and radiation suggest TREX1 inhibition would promote anti-cancer effects (40, 49, 51–53, 84). This concept is supported by studies showing that cells deficient in TREX1 activity show reduced recovery from treatment to DNA damaging agents (49, 84). Thus, inhibition of TREX1 in combination with chemotherapy may increase efficacy. Additionally, TREX1 functions to degrade DNA in dying cells (10, 50) and inhibition of TREX1 in tumor cells should potentiate the innate immune anti-tumor effect as these cells die during treatment. Thus, small molecules that inhibit TREX1 acting upstream of STING in the pathway could produce the added benefit to amplify the signal producing a more robust IFN-signal relative to the current, direct STING receptor-small molecule agonists. Furthermore, enzyme inhibitors are generally more easily developed and refined than activating molecules. Currently, STING agonists have demonstrated relatively poor pharmacokinetics and biodistribution, restricting their dosing routes primarily to intratumoral injection (70, 72, 80, 81, 85). By contrast, our work has identified several TREX1 inhibitors with good solubility and oral drug-like (86, 87) physicochemical properties (ex. compound discussed in **Figure 6**). Consequently, TREX1-targeted therapeutics have the potential to be administered through more convenient oral dosing routes and promote more robust, systemic antitumor immunity than their STING-targeted counterparts.

There are limited published data directly testing the effect of TREX1 ablation on antitumor immunity. In one study, human T lymphocytes derived from a *TREX1* compound heterozygote (c.262 ins AG het + c.290 g>a R97H het) with exonuclease-deficient enzyme exhibited an increased capacity to inhibit neuroblastoma cell growth *in vitro* (88), indicating the immunotherapeutic potential of TREX1 inhibition. It’s important to consider that acute TREX1 inactivation in wild-type organisms might not elicit the same biological response in *TREX1* mutants with chronic TREX1 inactivity. However, another study using microRNA-based TREX1-knockdown successfully demonstrated tumor regression *in vivo* (89), and in two additional studies it was shown that microRNA-based TREX1-knockdown generates an IFN signature in uninfected wild-type cells [see control data in refs (90, 91)]. Interpretation of these data is complicated by the complete loss of TREX1, including the TREX1 C-terminal region not required for exonuclease activity. However, we also observe that WT mice still produce IFN signatures when they receive bone-marrow transplants from mice with catalytically-inactive enzyme (*TREX1^D18N^* mice), though to a lesser degree than seen in the donors (37). These bone marrow transplants do not perfectly represent an acute induction of TREX1 dysfunction in the recipients, since the donor cells still developed in an environment of chronic TREX1 deficiency. Still, together these studies support the immunotherapeutic potential of acute TREX1 inhibition.

**Figure 3:**
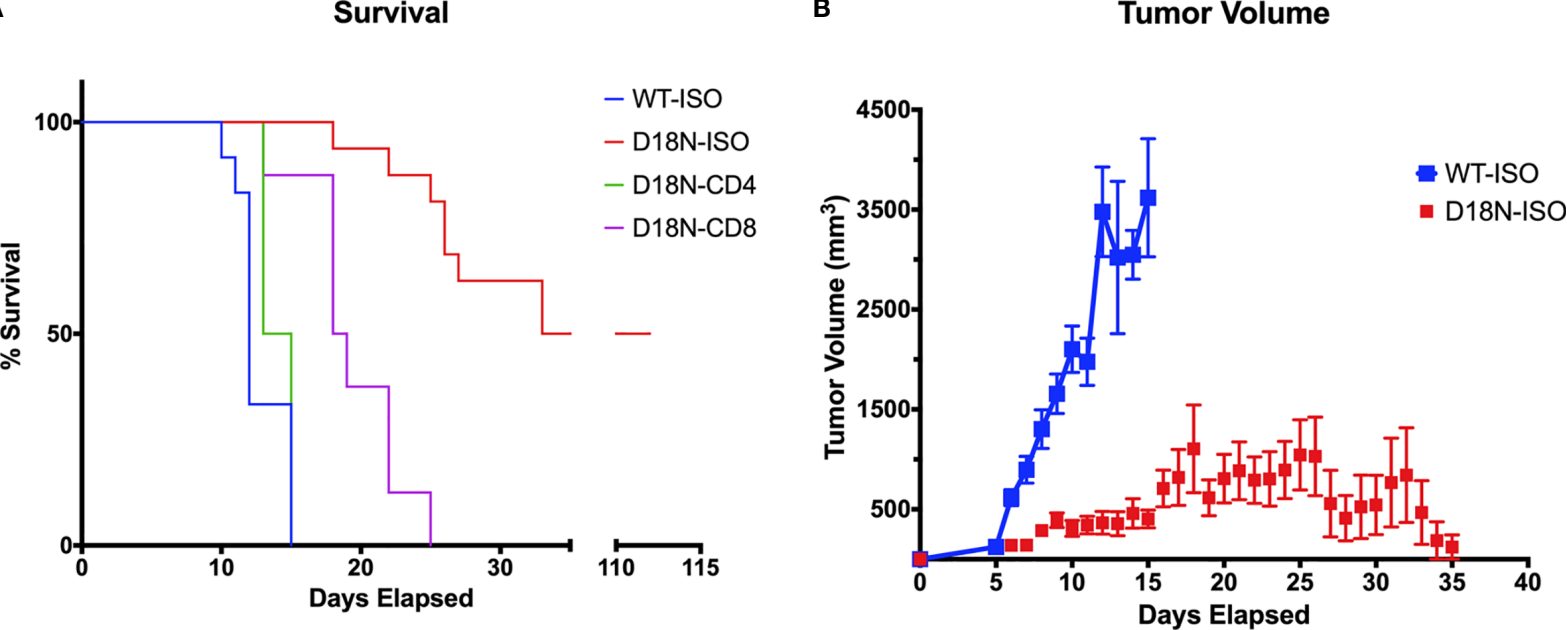
TREX1^D18N^ Mice Display T-cell Dependent Antitumor Immunity. **(A, B)** 5x10^6^ H31m1 tumor cells were injected subcutaneously into WT and D18N mice, and survival **(A)** and tumor volume **(B)** tracked daily (see Methods). Mice were treated with αCD4, αCD8, or the respective isotype-control antibodies to test the effects of T-cell depletion (see Methods). Isotype controls are presented together. Tumor volumes are average and standard deviation. Background of mice and tumor cells was 129S1/SvImJ, and each group represents 8-16 mice across 2-4 independent experiments. Data originally submitted for ASBMB 2020 conference (93). Graphs generated with Prism 7.0 (GraphPad).

The Perrino lab used allelic replacement to introduce the TREX1 D18N missense mutation into mice and showed that the TREX1 D18N mutation exhibits dysfunctional dsDNA-degrading activity resulting in immune activation in these mice (92). We tested the anti-cancer therapeutic potential of abolishing TREX1 exonuclease activity using the genetically precise TREX1^D18N^ mice (D18N mice), that express the mouse TREX1 D18N allele from its endogenous promoter that controls the level of expression in the appropriate genomic context. In this mouse model, the TREX1^D18N^ enzyme maintains structure, localization, and presumably protein-protein interactions making it an excellent model of specific inhibition of TREX1 exonuclease activity (7, 16, 92). The specific D18N mutation locates to the TREX1 active site in a way that TREX1 inhibitors might also bind and inhibit TREX1 DNA degradation, making the D18N mouse an appropriate model for TREX1 inhibition. We measured tumor resistance in the exonuclease deficient D18N mice by challenging WT and D18N mice with H31m1, a syngeneic, chemically induced sarcoma. When 5x10^6^ H31m1 cells were implanted subcutaneously in TREX1 WT mice, the tumor grew until one axis extended past 20 mm and mice were euthanized. In WT mice median survival was 13 days (**Figure 3A**). In sharp contrast, the inactivity of TREX1 exonuclease in the TREX1 D18N mice resulted in a dramatically reduced tumor volume that was always ~10-fold less than WT and an equally dramatic increase in median survival that was extended from 13 to 78 days in long-term survival studies, with ~40% of animals cured of their tumor for at least 120 days (**Figure 3B**).

**Figure 4:**
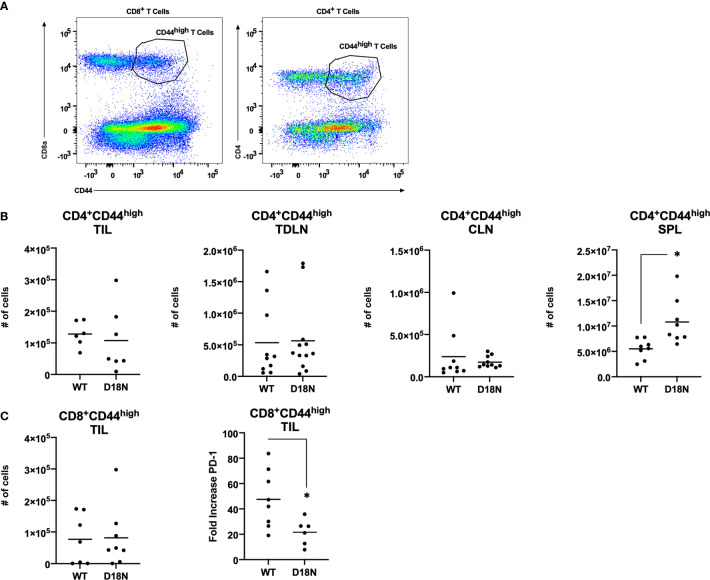
Similar T-Cell Number but Decreased PD-1 Expression in TREX1^D18N^ Mice During Tumor Progression. **(A–C)** WT or D18N mice were challenged with 5x10^6^ H31m1 cells, cells were isolated from the indicated tissue on Day 8, and **(A)** activated/memory CD4^+^ and CD8α^+^CD44^high^ T-cells were measured by flow cytometry (see Methods). Numbers of indicated **(B)** CD4^+^ or **(C)** CD8+ T cells were determined. ‘SPL’ = spleen, ‘CLN’ = contralateral lymph nodes, ‘TDLN’ = tumor draining lymph node, and ‘TIL’ = tumor infiltrating lymphocytes. **(C)** PD-1 M.F.I. on activated/memory CD8^+^CD44^high^ T-cells in the tumor were determined, and the fold change compared to naïve T-cells in the spleen was calculated (see Methods). Individual mice (6-9 total, 3 independent experiments) are plotted, with averages represented by horizontal bars. *p-value < 0.05 *via* two-tailed independent Student’s t-test. All graphs prepared in Prism 9.0 (GraphPad).

Further studies were done to identify the mechanisms that control tumor growth. Mice were pretreated with antibody specific for murine CD4 (to eliminate helper T cells) or CD8 (to eliminate cytolytic T cells) or with the isotype controls in a depletion analysis. Isotype-treated TREX1 D18N mice had significantly reduced tumor growth and longer median survival of 78 days sharply contrasting the TREX1 WT mice treated with control antibodies that had rapid tumor growth and a median survival time of only 13 days (**Figure 3A**). We have also examined the immune response in the spleen, contralateral lymph node (CLN), draining brachial and axillary lymph nodes (TDLN), and tumor infiltrating lymphocytes (TIL) using multidimensional flow cytometry for multiple cell types in the adaptive immune response in order to determine how the anti-tumor response is altered in TREX1 D18N mice. **Figure 4A** shows an example of T cells staining in the spleens of WT mice. Similar numbers of activated CD4^+^CD44^high^ T cells were observed in the TIL (**Figure 4B**), TDLN (**Figure 4B**), CLN (**Figure 4B**), whereas the spleens of TREX1 D18N animals had increased effector/memory CD4^+^ T (**Figure 4B**). When CD8+ T cells similar trends were also observed (**Figure 4C**). The most dramatic difference observed is in the fold induction in PD-1 levels on activated CD8^+^CD44^high^ T cells in the tumor (compared to CD8CD44^low^ T cells in the spleen). Here we observed that WT CD8^+^ T cell had a 40-fold induction of PD-1 levels (as measured by Mean Fluorescent Intensity (MFI)) whereas in TREX1 D18N mice levels were only increased ~20-fold, consistent with lower T cell exhaustion (**Figure 4C**). Taken together these results argue that the increased long-term survival observed after tumor challenge in TREX1 D18N mice is potentially due to altered function of CD4^+^ and CD8^+^ T cells. These studies support enhanced tumor immunity in the D18N mice, and TREX1 inhibition as a viable anticancer immunotherapeutic strategy.

## Methods of TREX1 Inhibition

Newly developed therapeutics are typically one of three general categories: RNA-based drugs (RBDs), biologics (BLGs), or small molecules (SMs). The advantages and disadvantages of these various molecules as therapeutics have been previously reviewed (94). RBDs are a variety of specifically designed RNA molecules that modulate the activity of a protein target by interfering with its translation (95, 96). It is possible that an RBD strategy targeting TREX1 and resulting in complete ablation of TREX1 protein could generate a successful immune activation anti-cancer effect. Indeed, it has been demonstrated that microRNA targeting of *TREX1* expression successfully promotes tumor regression *in vivo* by modulating the tumor microenvironment (89). However, we, and others, have demonstrated that it is elimination of TREX1 exonuclease activity specifically that leads to cGAS-STING pathway activation (92, 97). The complete removal of TREX1 protein using RBD could potentially impact, unnecessarily, additional TREX1 functions that are independent of the cGAS-STING pathway (8, 9). In fact, mutations that completely abolish TREX1 protein generally produce much more severe phenotypes in mice and humans than those specifically affecting the exonuclease activity (18, 19, 92). BLGs are a diverse category of mostly proteinaceous biologic macromolecules capable of target-binding (94, 98). Currently, there are no BLG inhibitors of TREX1 reported. However, several antibodies for TREX1 are commercially available (99), though not as neutralizing/inhibiting molecules. SMs are organic molecules with molecular weights typically in the 100-1000 Daltons range (86). SMs modulate the activity of a protein target *via* a direct and limited binding interaction, an approach most compatible with attenuation of a specific activity for multifunctional targets. Also, SMs have been quite successful as cancer immunotherapeutics (100), prompting our choice for designing TREX1 inhibitors (93).

## Developing Small Molecule TREX1 Inhibitors

A high-throughput screening (HTS) strategy is critical to identifying candidate small molecule TREX1 inhibitors. We have reported methodology to successfully purify large quantities of recombinant TREX1 enzyme (101) to facilitate a scalable biochemical assay. Since the desired therapeutic effect from TREX1 inhibition is cGAS stimulation (26, 27, 97, 102) and because cGAS has specificity for dsDNA (103), TREX1 exonuclease activity on dsDNA is the appropriate biochemical metric for an inhibitor’s potential. We have described a fluorescence-based exonuclease assay to measure TREX1’s degradation of dsDNA (101) that is scalable to a 384-well microplate HTS assay. Importantly, this assay utilizes substrate concentrations at or below the TREX1 dsDNA K_m_ of ~15 nM^2^, allowing the assay to readily detect small molecules with a broad range of inhibition kinetics (104). In addition, our own work has shown that TREX1 activity is not impacted by concentrations of up to 0.01% Triton X-100, which could be included in a HTS to limit false-positives from promiscuous aggregation-based inhibitors (105–107). Thus, our TREX1 biochemical studies have positioned us well to undertake a HTS endeavor.

**Figure 5:**
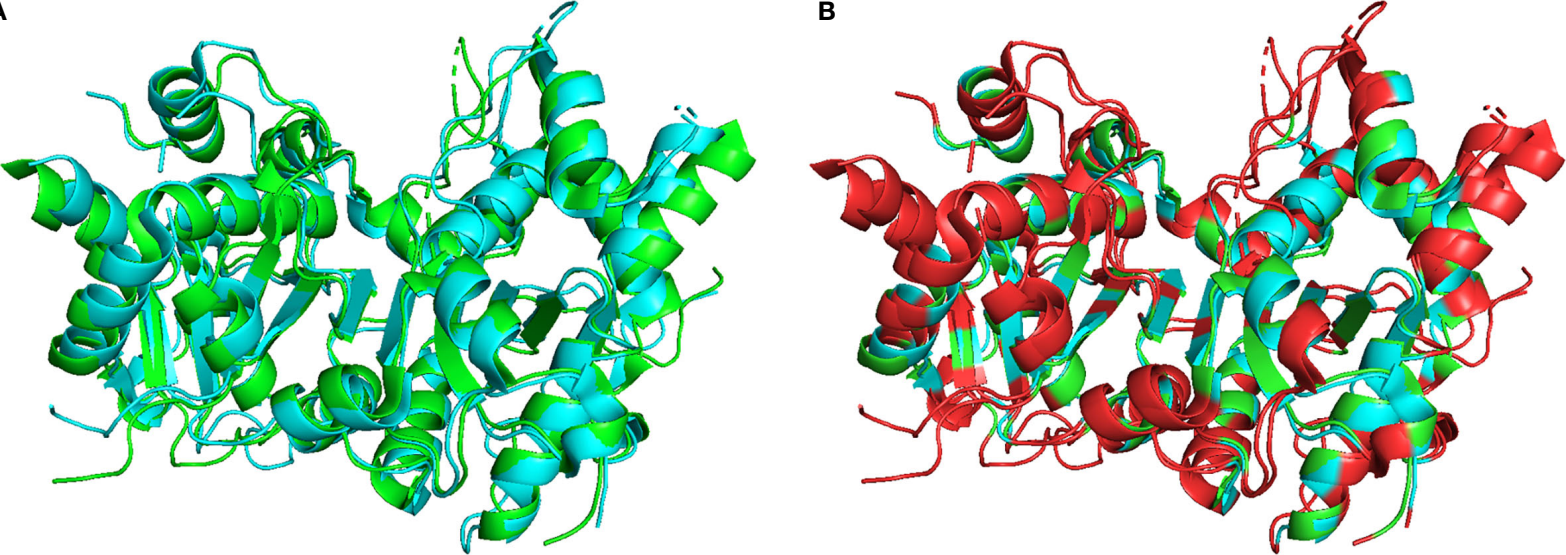
Structural Comparison of TREX1 and TREX2. Graphic **(A)** shows structural alignment of mTREX1(1-242) and hTREX2 in cyan and green, respectively, and graphic **(B)** is the same alignment with discrepant residues colored red. Alignment and graphics were generated in PyMOL using the PDB structures 3MXJ and 1Y97 from refs (16, 108).

**Figure 6:**
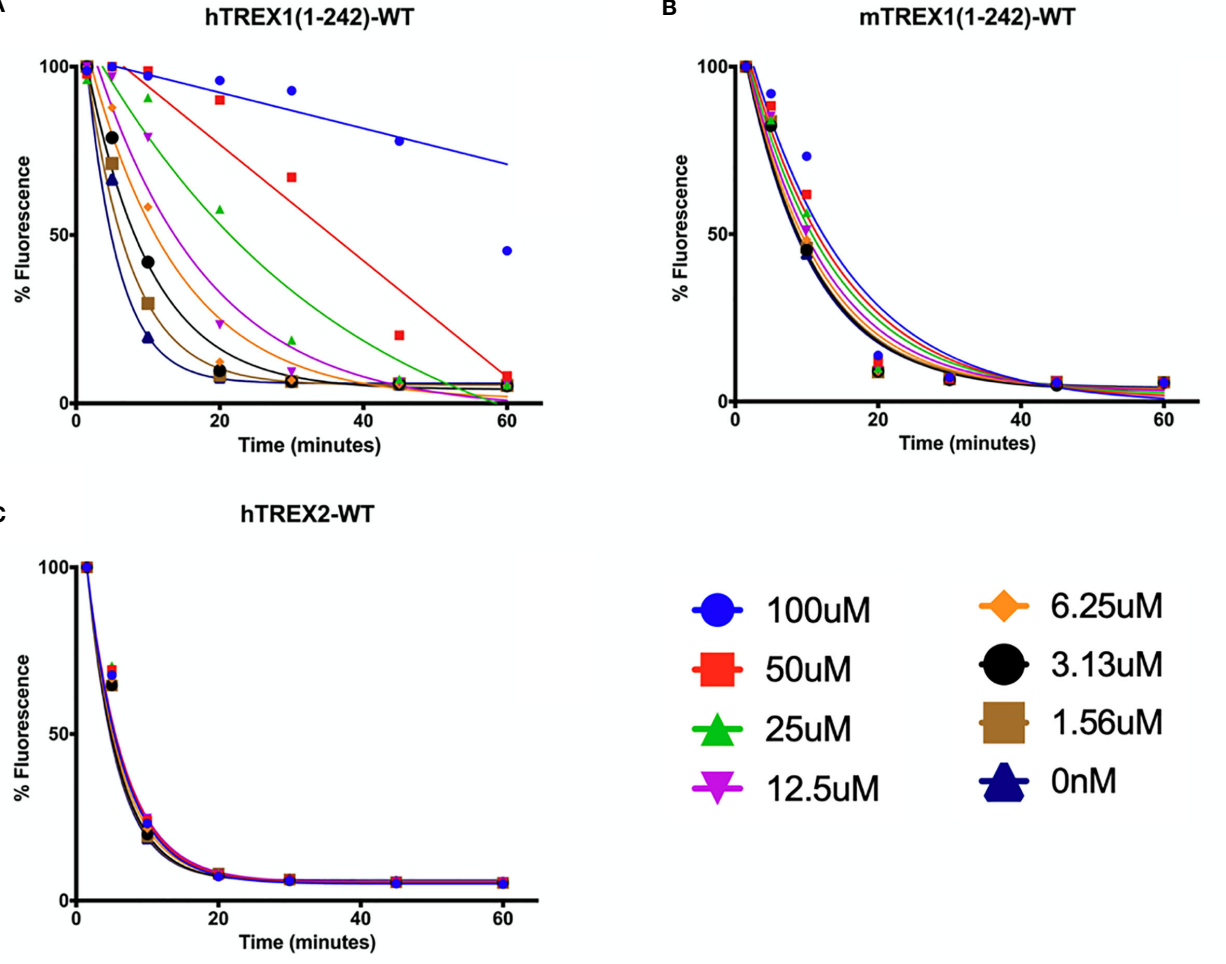
Small Molecule Inhibitor with High Specificity for hTREX1. **(A–C)** Standard time-course reactions were prepared in 150 μL volumes containing vehicle or indicated concentrations of inhibitor, and hTREX1 **(A)**, mTREX1 **(B)**, or hTREX2 **(C)**. Reactions were incubated 1-hr at room temperature, and 20 μL samples of each reaction taken at time-points of 0, 5, 10, 20, 30, 45, & 60 minutes and quenched in 20 μL of 15X SYBR Green. Fluorescence was measured, and fluorescence vs time plots were normalized to maximal initial fluorescence and background fluorescence (see Methods). Plots were fit with nonlinear regression. Plots were generated in Prism (GraphPad) and combined in PowerPoint.

Optimal TREX1 drugs developed from initial inhibitor molecules should minimize off-target effects and exhibit a high level of specificity for the target. Counter-screening candidate TREX1 inhibitors against enzymes of varying relatedness (104) provides context for the inhibitors’ relative affinities for the target. Cross-activity on a highly unrelated enzyme might suggest significant promiscuity by the candidate molecule, while inactivity against an enzyme likely indicates a level of specificity proportional to the enzyme’s relatedness to the target. Three-prime Repair Exonuclease 2 (TREX2) is structurally (6, 108) and biochemically (2) related to TREX1, making it the ideal choice for counter-screens to identify highly specific TREX1 inhibitors. Indeed, the similarities between TREX1 and TREX2 raise concerns about the potential for off-target effects *in vivo*, since TREX2 dysfunction has been linked to skin carcinogenesis in mice (109). However, TREX2 mutant mice exhibit a conditional phenotype requiring genotoxic stress (109, 110), suggesting that some level of TREX2 cross-activity by a TREX1 inhibitor might be tolerable for therapeutic applications. Despite the remarkable structural similarities, TREX1 and TREX2 contain multiple different structural elements and specific residues that could be exploited as TREX1-inhibitor contacts to achieve specificity (**Figure 5**). In addition, the potential for species specificity of small molecules, as evidenced by the STING agonist DMXAA’s ability to activate murine but not human protein (83), indicate biochemical analysis of human and mouse TREX1 to be a valuable approach. Our work using human and mouse TREX1 and TREX2 has already led to the identification of a class of small molecules with exceptional specificity for the hTREX1 enzyme (**Figure 6**).

**Figure 7:**
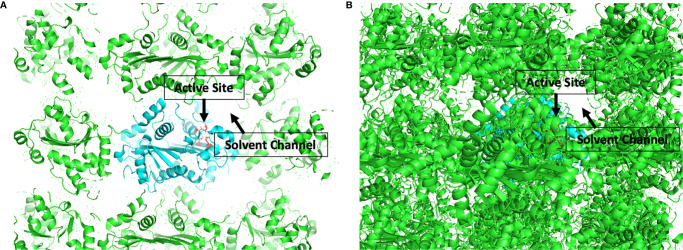
Active Sites are Accessible by Solvent Channel in TREX1 Apoenzyme Crystals. Structural representation of crystal lattice for mTREX1(1-242) apoenzyme crystal. Functional unit of interest is colored cyan with DEDD active site residues for one protomer shown as red sticks; other functional units are colored green. Graphic **(A)** is a slice through the crystal lattice where the active site is visibly facing the solvent channel, and graphic **(B)** looks through the solvent channel into the crystal lattice. Alignment and graphic were generated in PyMOL using the PDB structure 3MXJ from ref (16).

The development of candidate inhibitors identified from initial screening into effective therapeutics requires iterative chemical modification and testing to improve potency and specificity. This process benefits significantly from ‘rational’ design of the chemical modifications, which relies heavily on structural information about the target-inhibitor interactions. In this capacity, TREX1 is well suited for rational drug design. We have published a detailed protocol for generating large quantities of high purity TREX1 enzyme (101) and multiple structures of the mTREX1 enzyme solved by x-ray diffraction (6, 11, 16, 92), including apoenzyme and co-crystallizations with TREX1 substrates and product. These structures demonstrate the capacity for mTREX1 to be co-crystallized with a variety of molecules and can also be used in computational approaches to model the binding mechanisms of candidate inhibitors (111, 112). Our previously published mTREX1 apoenzyme indicates an active-site readily accessible *via* solvent channels in the crystal (**Figure 7**), suggesting TREX1-inhibitor co-structures could be determined by soaking compounds into existing apoenzyme crystals. We have also solved several structures of the hTREX2 enzyme (108, 113). Thus, crystallographic studies with the TREX2 enzyme present an alternative strategy to deduce target interactions in TREX1 inhibitors exhibiting cross reactivity. Altogether, our structural studies lay the groundwork for rational design modifications that contact residues discrepant between the two enzymes.

## Summary

The 3’ → 5’ exonuclease TREX1 acts *in vivo* to degrade DNA and prevent aberrant nucleic acid sensing. In the absence of TREX1 exonuclease activity, substrate accumulation stimulates the DNA-sensing pathway cGAS-STING, which drives IFN-signaling and autoimmunity. TREX1 and cGAS-STING have been proposed to function in the tumor microenvironment, where TREX1 is believed to degrade tumor-derived DNA that would otherwise stimulate the cGAS-STING pathway and elicit antitumor immunity. Thus, we propose TREX1 as a novel immunotherapeutic target, and provide data demonstrating significant antitumor immunity in TREX1-deficient mice. We propose small molecules as a viable strategy for TREX1 inhibition in the context of past work with other targeting strategies.

## Materials and Methods

### Expression and Purification of TREX Enzymes

Detailed protocols for hT1, mT1, and hT2 enzyme purification have been published (101) and summarized here. For homodimers, pLM303x constructs encoding the recombinant TREX enzyme fused to an N-terminal maltose-binding protein (MBP) are transformed into Rosetta II cells, and the MBP-linker-TREX fusion protein is overexpressed. Cells were pelleted, subjected to pressure-lysis, and the supernatants subjected to amylose column chromatography. Eluent was treated with protease to cleave the fusion protein linker and purified by phosphor-cellulose column chromatography to obtain pure TREX enzyme.

### Fluorescence-Based Exonuclease Assay

Our detailed protocol for this assay is published (101). Reaction mixture was prepared containing variable concentrations of a dsDNA substrate, 5 mM MgCl_2_, 2 mM DTT, 20 mM Tris base (pH 7.5). Compounds were added at various concentrations as DMSO solutions to the reaction mixture prior to enzyme addition, and final DMSO-vehicle concentration is 2.5% in all exonuclease experiments. Enzyme was diluted to 10X the final reaction concentration *via* serial dilutions into 1 mg/mL BSA, and then diluted 10-fold into the reaction to initiate resulting in the appropriate TREX enzyme and BSA at 100 µg/mL. Reactions were at room-temperature for 1hr. Samples (20 µL) were removed at varied time points and quenched in a 384-well black microplate containing 20 µL of 15X SYBR Green solution. Fluorescence of quenched samples was measured using a PolarStar Omega microplate reader (BMG LabTech) at excitation/emission of 497/520.

The DNA substrate was generated by linearizing the ~10-kb pMYC plasmid with the SacI (NEB) restriction enzyme per vendor specifications and included in assays at a concentration of 5 ng/µL. Enzyme concentrations were 15 nM for mT1, 75 nM for hT2, and 15 nM for hT1. Time-course reactions were from 20 µL samples taken at 0, 5, 10, 20, 30, 45, & 60-minute time points. Initial reaction volumes were 150 µL, compound concentrations were as indicated in the figure, and vehicle control reactions were always included.

### Tumor Challenge

Nine week 129 S6/SvEvTac D18N mutant and WT mice were generated as previously described (92). At 10 to 12 weeks, 5 × 10^6^ H31m1 tumor cells were subcutaneously (s.c) injected in 200 µl PBS into the shaved right flanks of recipient mice. Tumor size was measured by a digital caliper every day and presented as the cube of its diameters. Studies included 6-9 mice/group across 3 independent experiments. H31m1 cells were obtained from Robert Schreiber (Washington University). All studies were approved by the Institutional Animal Care and Use Committee (IACUC) of the Wake Forest University School of Medicine.

### Antibody Depletion Experiments

To determine which cells were essential for enhanced clearance in D18N mice, we depleted CD4^+^ or CD8^+^ T cells by administering 1500 µg of antibody (BioXCell) for 3 days prior to and during tumor challenge (-2, 0, + 2, i.p.). This resulted in 99% selective depletion as assessed by flow cytometry on PBMCs isolated at day 10. Clones 53-6.7 and GK1.5 were used for CD8α^+^ and CD4^+^ T cells, respectively.

### Cell Isolation

The spleen was removed from mice after cervical dislocation. Following mechanical disruption of splenocytes on a wire mesh screen, red blood cells were removed by osmotic lysis in ACK buffer (NH4Cl, KHCO3, and EDTA). Splenocytes were then resuspended in complete media containing RPMI 1640 supplemented with 10% fetal calf serum (FCS, HyClone), L-glutamine (HyClone), penicillin-streptomycin (Cellgro), non-essential amino acids (GIBCO), and 2-mercaptoethanol (GIBCO). For CD8^+^ T cell purification, splenocytes were resuspended in PBS supplemented with FCS and EDTA. CD8^+^ T cells from splenocytes were then negatively selected by magnetic bead using CD8^+^ T-Cell Purification Kit (Miltenyi Biotec) according to the manufacturer’s instructions.

In tumor studies, the contralateral and draining lymph nodes (brachial and axillary) were isolated. Tumor tissues were dissociated by mechanical disruption and incubated with enzymes in Tumor Dissociation Kit (Miltenyi Biotec) at 37°C for 30 mins. TILs were then washed with RPMI 1640 supplemented with 1% fetal calf serum, and resuspended in complete media. Tumor analyses used whole tumors of approximately equivalent volume that were taken at 8-days post challenge. Cell numbers in **Figure 4** were determined by flow cytometry by gating on either CD8 or CD4, then CD44 (as shown in **Figure 4A**) and the PD-1 mean was calculated for CD8^+^CD44^high^ T-cells.

### Surface and Intracellular Staining

In this study, the following antibodies were used: rat anti-mouse CD8α-phycoerythrin (PE), rat anti-mouse CD8α-peridinin chlorophyll protein (PerCP), rat anti-mouse CD8α-V500, rat anti-mouse CD90.1 (Thy1.1)-allophycocyanin (APC), rat anti-mouse CD90.1- fluorescein isothiocyanate (FITC), rat anti-mouse CD90.1-eFluor450, rat anti-mouse CD90.2 (Thy1.2)-V500, rat anti-mouse CD4-APC, rat anti-mouse CD4-V500, rat anti-mouse CD44-PerCP, rat anti-mouse CD44-eFluor450, rat anti-mouse CD44-APC-eFluor780, rat anti-mouse CD127-FITC, rat anti-mouse KLRG1-PE, rat anti-mouse CD27-PE-Cyanine7, rat anti-mouse CD62L-APC-eFluor780, rat anti-mouse CD69-PE-Cyanine7, rat anti-mouse PD-1-FITC, rat anti-mouse LAG-3- PerCp-eFluor710, rat anti-mouse BTLA- PE, rat anti-mouse IFN-γ-FITC, rat anti-mouse TNF-α-PE-Cyanine7, rat anti-mouse IL-2-APC, rat anti-mouse CCL3 (MIP-1α)-PE. KLRG1 antibody was purchased from Abcam. CD8-V500, CD8-PerCp, IFN-γ-FITC, TNF-α-PE-Cyanine7 and IL-2-APC were purchased from BD Pharmingen. All other antibodies were purchased from eBioscience. Surface staining was performed by incubation of Abs at a 1:100 dilution in fluorescence-activated cell sorter (FACS) buffer for 30 min on ice. KLRG1 staining was performed at a 1:25 dilution. Tetramer staining was performed at a 1:200 dilution. BTLA staining was performed at a 1:333 dilution. To measure intracellular cytokine levels, cells were incubated with 50 ng/ml phorbol 12-myristate 13-acetate (PMA) and 500 ng/ml ionomycin (ION) for 5 h at 37°C, and then treated with the BD Biosciences Cytofix/Cytoperm kit according to the manufacturer’s instructions. Intracellular transcription factor stain was performed by using eBioscience Mouse Regulatory T Cell Staining Kit according to the manufacturer’s instructions. After staining, samples were fixed in 1% formaldehyde (Polysciences, lnc., Warrington, PA) and acquired on a BD FACS Canto instrument. Manual gating was performed on FlowJo software (TreeStar, San Francisco, CA).

## Data Availability Statement

The original contributions presented in the study are included in the article/supplementary material. Further inquiries can be directed to the corresponding author.

## Ethics Statement

The animal study was reviewed and approved by Animal Care and Use Committee at Wake Forest School of Medicine.

## Author Contributions

WH provided the inhibitor data and prepared the manuscript. SS and ML contributed to the murine tumor data and assisted with manuscript revisions. FS contributed to the inhibitor data and assisted with manuscript revisions. TH contributed to the inhibitor data and assisted with manuscript revisions. JG provided the murine tumor data and assisted with manuscript revisions. FP ideated the proposal and assisted with manuscript revisions. All authors contributed to the article and approved the submitted version.

## Funding

This work was supported by NIH (R01AI116725 to FP, T32GM095440 and T32AI007401 to WH), the Wake Forest Department of Biochemistry (Sandy Lee Cowgill memorial scholarship and Artom fellowship to WH), and a Scott Family Fellowship to FS.

## Conflict of Interest

WH, TH, and FP declare the filing of U.S. Provisional Application No. 62/706,167 Trex1 Inhibitors and Uses Thereof.

The remaining authors declare that the research was conducted in the absence of any commercial or financial relationships that could be construed as a potential conflict of interest.
